# Carbon Ion Irradiated Neural Injury Induced the Peripheral Immune Effects *in Vitro* or *in Vivo*

**DOI:** 10.3390/ijms161226109

**Published:** 2015-11-30

**Authors:** Runhong Lei, Tuo Zhao, Qiang Li, Xiao Wang, Hong Ma, Yulin Deng

**Affiliations:** 1 School of Life Science, Beijing Institute of Technology, Beijing 100081, China; leirh0581@gmail.com (R.L.); zhaotuobeijing@hotmail.com (T.Z.); 2 Department of Space Radiobiology, Key Laboratory of Heavy Ion Radiation Biology and Medicine, Institute of Modern Physics, Chinese Academy of Sciences, Lanzhou 730000, China; liqiang@impcas.ac.cn; 3 Department of Nuclear Physics, China Institute of Atomic Energy, Beijing 102413, China; junmei33@aliyun.com

**Keywords:** carbon ions, neural injury, peripheral immune system, side effects, T-cell development

## Abstract

Carbon ion radiation is a promising treatment for brain cancer; however, the immune system involved long-term systemic effects evoke a concern of complementary and alternative therapies in clinical treatment. To clarify radiotherapy caused fundamental changes in peripheral immune system, examinations were performed based on established models *in vitro* and *in vivo*. We found that brain-localized carbon ion radiation of neural cells induced complex changes in the peripheral blood, thymus, and spleen at one, two, and three months after its application. Atrophy, apoptosis, and abnormal T-cell distributions were observed in rats receiving a single high dose of radiation. Radiation downregulated the expression of proteins involved in T-cell development at the transcriptional level and increased the proportion of CD3^+^CD4^−^CD8^+^ T-cells in the thymus and the proportion of CD3^+^CD4^+^CD8^−^ T-cells in the spleen. These data show that brain irradiation severely affects the peripheral immune system, even at relatively long times after irradiation. In addition, they provide valuable information that will implement the design of biological-based strategies that will aid brain cancer patients suffering from the long-term side effects of radiation.

## 1. Introduction

Owing to the physical properties of carbon ion beams, carbon ion radiotherapy is preferentially recommended for treatment of brain cancers [1,2]. Compared with other radiotherapies, it has a higher relative biological effectiveness, particularly at the distal edge of the Bragg peak which may improve tumor control, and a smaller lateral penumbra, which may allow more conformal lateral doses and better limit normal tissue damage [3,4]. Cranial radiotherapy is an effective treatment for primary and metastatic brain tumors [5]. Advances in treatment planning and delivery have made it possible to safely deliver a small number of high doses (15–20 Gy) rather than many small doses of 1.8–2 Gy to tumors over the course of several weeks [6]. However, a single high dose can cause severe acute and long-term immune system involved systemic effects [7,8,9,10,11] that can enhance or suppress tumor metastasis [12,13,14] and conduce toward increased morbidity of other diseases [15,16]. However, studies about the side effects in the immune system have primarily concentrated on non-brain tumor radiotherapy and fractionated dose delivery [17,18,19,20,21,22,23] while the fundamental knowledge about the response in peripheral immune system after brain-localized single high-dose heavy ion radiotherapy has been largely overlooked. Moreover, previous studies focused on acute responses [24,25,26], despite the persistence of systemic effects modulated by immune system usually last for several or more months after treatment completion [27,28]. This focus may reflect the difficulty of assessing long-term responses owing to the emergence of complex defense mechanisms over time or the overemphasis on acute side effects that cause physical damage.

Toward the goal of assessing long-term immune responses to radiation, we have addressed the effects of heavy ion irradiation-induced neural cell damage on the peripheral immune system, which plays a key role in maintaining and modulating abscopal effects and systemic effects. We previously established an *in vitro* co-culture system to investigate neurotoxin-mediated regulation of the proliferation of peripheral immune cells [29]. In the present study, we examined the long-term effects on the peripheral immune system, including the peripheral blood, thymus, and spleen, after neural cell injury caused by carbon ion radiation *in vitro* and *in vivo*.

## 2. Results

### 2.1. Carbon Ion-Irradiated Neural Cells Mediate Immune Effects in Vitro

**Figure 1:**
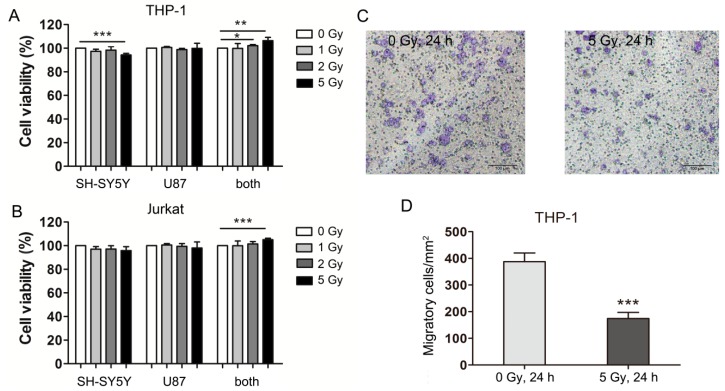
Carbon ion-irradiated neural cells mediate immune effects *in vitro*. (**A**) THP-1 cells and (**B**) Jurkat cells were cultured in three different types of conditioned medium and cell viability was determined 24 h later; (**C**) Representative images of THP-1 cells in transwells (bar = 100 μM); (**D**) Quantitation of THP-1 cell migration. SH-SY5Y and U87 cells alone and in combination were irradiated with carbon ions at a dose of 1, 2, or 5 Gy or were mock-irradiated (0 Gy). Medium conditioned by these cells was harvested 24 h after irradiation and transferred to THP-1 and Jurkat cells. SH-SY5Y, conditioned medium from SH-SY5Y cells; U87, conditioned medium from U87 cells; both, conditioned medium from co-cultured SH-SY5Y and U87 cells. * *p* < 0.05, ** *p* < 0.01, *** *p* < 0.001.

Previous reports showed that neural cells injury could enhance peripheral immune T cells’ proliferation and decrease monocytes migration and invasion [7,8,9,10,11]. In order to evaluate the immune effect induced by heavy ion irradiation and to simulate the *in situ* condition of the brain, neuron-like SH-SY5Y cells and astrocytic glial U87 cells were cultured together as described previously [29] and then irradiated with carbon ions (1, 2, or 5 Gy). The medium was collected 24 h after irradiation and applied to immune cells (human leukemia monocytic cell line THP-1 and human leukemic T cell line Jurkat). Medium conditioned by neural cells receiving 5 Gy of radiation increased the viability of both THP-1 and Jurkat cells compared with medium conditioned by mock-irradiated neural cells (Figure 1A,B). THP-1 cells are premonocytes that can be induced to differentiate into terminal macrophages. When exposed to the conditioned medium of neural cells receiving 5 Gy of irradiation, THP-1 cells differentiated into macrophages, which migrated to the lower surface of the membrane in transwell migration assays (Figure 1C). Significantly less THP-1 cells migrated when exposed to the conditioned medium of irradiated compared with mock-irradiated cells (Figure 1D). These data indicate that neural cell injury caused by carbon ion radiation may enhance both monocytes and peripheral immune T cells proliferation but decrease the migration and invasion of monocytes.

### 2.2. Carbon Ion-Irradiated Neural Cells Mediate Immune Effects in Vivo

**Figure 2:**
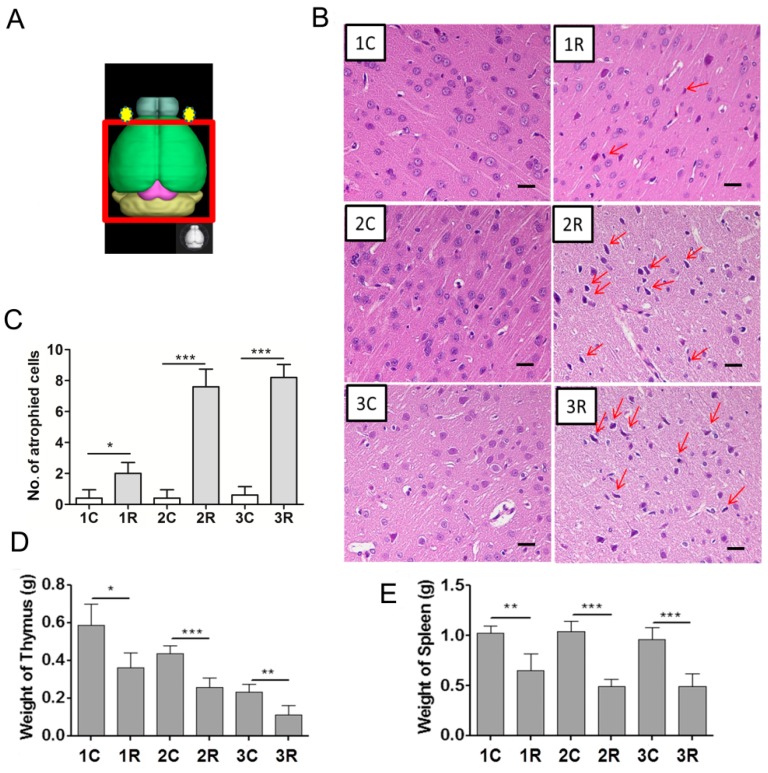
Brain-localized carbon ion radiation induces neural tissue injury and reduces the weight of the thymus and spleen. (**A**) Diagram of brain-localized radiation with carbon ions; (**B**) Hematoxylin and eosin staining of the parietal lobe cortex. Representative photographs from three rats are shown. The red arrows indicate specialized cells (bar = 20 μM); (**C**) Quantitative analysis of the specialized cells in the parietal lobe cortex; (**D**) Weight of the thymus at the indicated time points; (**E**) Weight of the spleen at the indicated time points. 1C, 2C, and 3C: control rats, one, two, and three months after mock irradiation, respectively. 1R, 2R, and 3R: irradiated rats, one, two, and three months after irradiation, respectively. * *p* < 0.05, ** *p* < 0.01, *** *p* < 0.001.

Since immune effects can be observed in heavy ions irradiated co-culture cell model, we focused on whether similar effects can be found *in vivo*. Firstly, we constructed the rat model of brain-localized carbon ion radiation (BLCIR). A high single-dose of 15 Gy was directed to three groups with seven rats in each group (the area exposed is shown in Figure 2A). One, two, and three months after irradiation, rats were sacrificed. Pathological changes in the central nervous system and effects on the immune organs (thymus and spleen) were measured. The most apparent differences in neural tissue after BLCIR were the increased number of cells surrounded by vacuoles and the appearance of darkly stained pantomorphic nuclei in the parietal lobe cortex (Figure 2B, red arrows). Numerous foci were observed two and three months after BLCIR, as shown in the representative photograph in Figure 2B. A quantitative analysis of the specialized cells (preferentially neurons undergoing atrophy) in the parietal lobe cortex is presented in Figure 2C, which implies the success of modeling. Secondly, thymus is important in the development of T-cells from hematopoietic precursors and maintenance of central tolerances, while the spleen is critical to removing dead red blood cells, recycling iron, removing antibody coated bacteria and cells, producing antibodies and serving as a reserve of monocytes and blood. Thus, we analyzed thymus and spleen indices including mass, which could partly reflect the status of the immune system in the rat BLCIR model. As shown in Figure 2D,E, both thymus and spleen weight decreased significantly in irradiated rats after BLCIR, although the age-associated atrophy of the thymus gland was noticed in control group. Moreover, this kind of decrease can be observed in a long term from one month to three months after heavy ion exposure. These data indicate that BLCIR effectively induces neural damage and decreases the mass of the thymus and spleen, and therefore demonstrate the usefulness of the BLCIR rat model.

### 2.3. BLCIR-Induced Apoptosis, Abnormality of T-Cell Development in Thymus

**Figure 3:**
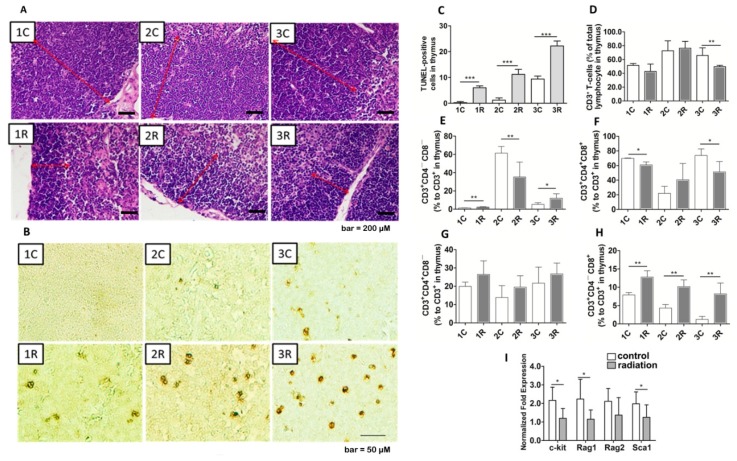
Shrinkage, cell apoptosis, and disordered T-cell development in the thymus of irradiated rats. (**A**) Thymic cortical thinning in irradiated rats. The red lines indicate the thymic cortex (bar = 200 μM); (**B**) Apoptotic cells in the thymus (bar = 50 μM); (**C**) Quantitation of terminal deoxynucleotidyl transferase dUTP nick end labeling (TUNEL)-positive cells in the thymus; (**D**) Proportion of CD3^+^ T-cells in the thymus; (**E**) Percentage of double-negative (CD4^−^CD8^−^) T-cells; (**F**) double-positive (CD4^+^CD8^+^) T-cells; (**G**) CD4^+^CD8^−^ T-cells; and (**H**) CD4^−^CD8^+^ T-cells. The number of cells in each subtype was normalized to the total number of CD3^+^ T lymphocytes. Each data point represents the results from seven rats; (**I**) mRNA levels of the proteins involved in T-cell development. The mRNA levels of the indicated proteins were normalized to the mRNA level of glyceraldehyde 3-phosphate dehydrogenase. Each data point represents the results from four rats. Representative photographs from three rats are presented in (**A**,**B**). 1C, 2C, and 3C: control rats, one, two, and three months after mock irradiation, respectively. 1R, 2R, and 3R: irradiated rats, one, two, and three months after irradiation, respectively. * *p* < 0.05, ** *p* < 0.01, *** *p* < 0.001.

Because BLCIR reduced the mass of the thymus, we examined its pathological effects and its effects on the distribution of T-cell subtypes. Hematoxylin and eosin staining showed thymic cortex thinning in cross-sections of irradiated rats compared with cross-sections of control rats taken from similar regions (Figure 3A, red lines). Time-dependent increases in the number of terminal deoxynucleotidyl transferase dUTP nick end labeling (TUNEL)-positive cells in the thymus were observed in control rats and, to a greater extent, in irradiated rats (Figure 3B,C). Thymic cell apoptosis increases with age [30], which presumably explains the increased number of TUNEL-positive thymic cells in the control rats. These findings suggest that promoted thymic involution in irradiated rats may result from a decrease in the number of thymocytes and an increase in the number of apoptotic cells.

After that, flow cytometry revealed that the proportion of CD3^+^ T cells was unaffected until three months after irradiation (Figure 3D). Sequential subtypes analysis showed that, irradiation caused significant changes in the percentages of double-negative (CD4^−^CD8^−^), double-positive (CD4^+^CD8^+^), and single-positive (CD4^+^CD8^−^, CD4^−^CD8^+^) T-cells in the thymus (Figure 3E–H). Interestingly, the percentage of thymic CD3^+^CD4^−^CD8^+^ T lymphocytes was higher in irradiated rats than in control rats at all three time points (Figure 3H). Most CD3^+^CD4^−^CD8^+^ T cells will mature and become cytotoxic upon activation by a class I-restricted antigen and will play an important role in immune response. Therefore, these data indicate that BLCIR impairs T-cell development and selection in the thymus.

We next focused on the genes involved in the regulation of T-cell development. C-kit, also known as CD117, is a *bona fide* marker of double-negative T-cells [31]. Rag1 and Rag2 are the lymphocyte-specific components of the V(D)J recombinase [32]. Sca-1 is expressed throughout T cell ontogeny and can subdivide thymic and peripheral T lymphocytes into unique subsets [33]. As determined by real-time PCR, the mRNA levels of c-kit, Rag1, Rag2, and Sca-1 were lower in the thymuses of irradiated rats than that of control rats (Figure 3I). Down-regulation of the transcription level of these genes represents a potential mechanism whereby BLCIR affects the peripheral immune system.

### 2.4. BLCIR Induced Apoptosis, T-Cell Distribution in Spleen

**Figure 4:**
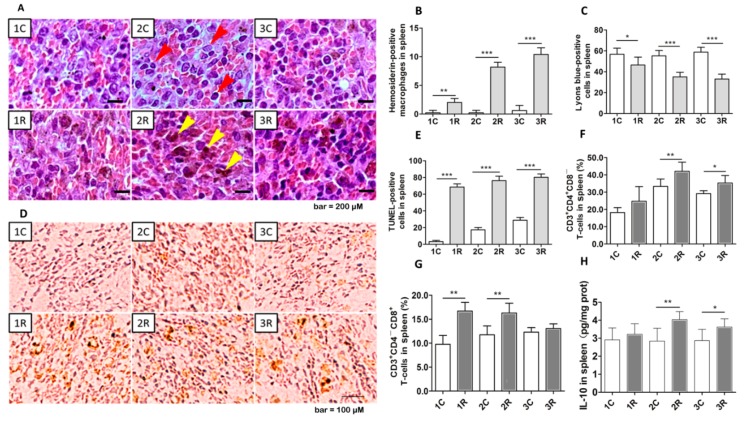
Apoptosis and distribution of T-cell subtypes in the spleen. (**A**) Hematoxylin and eosin staining of the spleen, red arrows indicate splenocytes with Lyons blue-stained nuclei and yellow arrows indicate reddish brown featured phagocytes (bar = 200 μM); (**B**) Quantitative analysis of hemosiderin-positive phagocytes; (**C**) Quantitative analysis of Lyons blue-positive cells; (**D**) Apoptotic cells evaluated via terminal deoxynucleotidyl transferase dUTP nick end labeling (TUNEL) (bar = 100 μM); (**E**) Quantitative analysis of TUNEL-positive cells; (**F**) Proportion of CD3^+^CD4^+^CD8^−^T-cells; (**G**) Proportion of CD3^+^CD4^−^CD8^+^ T-cells; (**H**) Level of interleukin 10 (IL-10). All parameters were measured in the spleen. Representative photographs from three rats are presented in (**A**,**D**). (**F**–**H**) Each time point represents the results from seven rats. 1C, 2C, and 3C: control rats, one, two, and three months after mock irradiation, respectively. 1R, 2R, and 3R: irradiated rats, one, two, and three months after exposure, respectively. * *p* < 0.05, ** *p* < 0.01, *** *p* < 0.001.

As described above, BLCIR substantially reduced the mass of the rat spleen. Then, pathological examination and quantitative analysis revealed increases in the number of hemosiderin-positive macrophages in the spleens of irradiated rats (Figure 4A,B, reddish brown cells indicated by yellow arrows), presumably due to enhanced phagocytosis of red blood cells and hemoglobin. Extracellular matrix (Figure 4A, dark red-brown staining) was more abundant in the spleens of irradiated rats than in those of control rats. Splenocyte density was reduced in irradiated rats compared with control rats at all three time points (Figure 4A,C; splenocytes have Lyons blue-stained nuclei and are indicated by red arrows in Figure 4A). The number of TUNEL-positive cells was significantly higher in the spleens of irradiated rats than in those of control rats at all the time points (Figure 4D,E). BLCIR increased the proportion of CD3^+^CD4^+^CD8^−^ and CD3^+^CD4^−^CD8^+^ T lymphocytes in the spleen, as determined via flow cytometry (Figure 4F,G), and the abundance of interleukin 10 (IL-10), an indicator of immunosuppression and an anti-inflammatory cytokine produced by the spleen (Figure 4H). These data suggest that BLCIR causes substantial splenic involution, cell apoptosis and increases the proportion of CD3^+^ T-cells.

### 2.5. BLCIR-Induced Immunosuppression Features in Peripheral Blood

**Figure 5:**
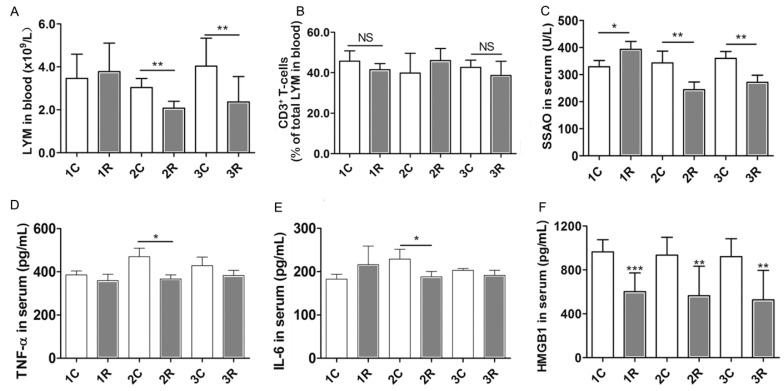
Immunosuppression in the peripheral blood after brain-localized carbon ion radiation. (**A**) Concentration of lymphocytes (LYM) in the peripheral blood; (**B**) Proportion of CD3^+^ T-cells in the peripheral blood; (**C**–**F**) Concentration of inflammation factors [semicarbazide-sensitive amine oxidase (SSAO), tumor necrosis factor-α (TNF-α), interleukin-6 (IL-6), and high mobility group box-1 (HMGB1) protein in serum]. Each time point represents the results from seven rats. NS: not significant. 1C, 2C, and 3C: control rats, one, two, and three months after mock irradiation, respectively. 1R, 2R, and 3R: irradiated rats, one, two, and three months after irradiation, respectively. * *p* < 0.05, ** *p* < 0.01, *** *p* < 0.001.

Lymphocyte concentration in the peripheral blood represents the defense capacity. We found that, lymphocyte concentration was significantly lower in the irradiated rats than in control rats two and three months after treatment (Figure 5A). However, the proportion of CD3^+^ T-cells in the peripheral blood was unaffected (Figure 5B). Irradiation generally reduced the serum concentrations of semicarbazide-sensitive amine oxidase (SSAO), tumor necrosis factor-α (TNF-α), interleukin 6 (IL-6), and high mobility group box-1 (HMGB1) protein, all of which are involved in inflammation (Figure 5C–F). SSAO facilitates leukocyte accumulation during inflammation, and HMGB1 levels increase in serum during sterile tissue injury and infection. These results indicate that BLCIR reduces lymphocyte concentration and the abundance of factors involved in inflammation. These changes were evident even three months after irradiation.

## 3. Discussion

Radiation of brain cancers induces chronic systemic changes, primarily by perturbing the immune system [34]. Since these changes may enhance or suppress the growth of primary and secondary tumors, characterization of the long-term immune response after brain-localized heavy ion radiation is important for health maintenance and tumor control. In this study, we evaluated the immune effect mediated by neural radiation injury *in vitro* and the peripheral immune responses in rats one, two, and three months after BLCIR *in vivo* to determine how it affects the immune system and consequently systemic events.

Neural tissue injury may induce immune responses via multiple systems including the hypothalamic-pituitary-adrenal (HPA) axis-based neuroendocrine regulation network, the sympathetic nervous system and the central nervous system lymphatic vessels; cyclic secretions of inflammatory cytokines, chemokines, growth factors, and small molecules may also be involved. Cerebrospinal fluid, as a buffer of signaling molecules, its drainage into periphery could potentially mediate the dysfunction in peripheral immune organs [35,36]. BLCIR did not significantly affect the concentrations of corticotrophin-releasing hormone and adrenocorticotropic hormone in the peripheral blood in our study (Supplementary Figure S1). This finding indicates that neural injury induces long-term peripheral immune responses independently of HPA axis-associated hormonal pathways. Similar findings were reported [6,37,38]. Our finding that the conditioned medium of irradiated neural cells promotes the proliferation of immune cells suggests that soluble factors (e.g., cytokines and chemokines) released by neural cells mediate the communication between the central nervous and immune systems. This premise agrees with the results of others [39,40,41]. Notably, the effectiveness of the conditioned medium depended on a combination of neural cells and the types of immune cells targeted.

Previous studies have shown that the thymus is susceptible to whole body irradiation [42,43,44,45]. We found that the thymus was also sensitive to BLCIR, as demonstrated by atrophy, thymic apoptosis, and abnormal T-cell development in rats receiving BLCIR. Although glucocorticoid receptor (GR) signaling usually controls thymocyte homeostasis [46,47,48,49], recent studies suggest that, thymic apoptosis can be triggered by various stimuli independent of glucocorticoids (GCs) [38,50]. In our rat model, levels of GCs and GRs in the thymus were significantly elevated two months after irradiation, but not at one or three months (Supplementary Figure S2); elevation at two months may be due to age-associated thymic development [51,52]. Hormone-dependent and GC-independent pathways may both regulate thymic homeostasis, although at different stages of thymic development and involution, perhaps resulting in imbalanced homeostasis and increased development of CD3^+^CD4^−^CD8^+^ T lymphocytes in the thymus. CD3^+^CD4^−^CD8^+^ T lymphocytes mainly become cytotoxic T lymphocytes and thus may play a vital role in maintaining basic immune function after irradiation.

As previously reported, the splenic sympathetic nerve can release neurotransmitters, such as acetylcholine, norepinephrine, epinephrine, and neuropeptide Y, all of which control innate immune responses [53,54,55]. Release of neurotransmitters may occur in rats receiving BLCIR. Notably, irradiation-induced increases in T-cell viability (as shown for Jurkat cells *in vitro*) and changes in the proportions of CD3^+^CD4^+^CD8^−^ and CD3^+^CD4^−^CD8^+^ T-cells (as shown *in vivo*) may aid in controlling tumor growth [56]. We suggest that CD3^+^ T-cells in the blood and CD3^+^CD4^+^CD8^−^ and CD3^+^CD4^−^CD8^+^ T-cells in the spleen are not vulnerable but rather are activated in response to BLCIR. Irradiation also triggered THP-1 proliferation and differentiation, as assessed by measuring cell viability and migration in the co-culture system. However, other types of immune cells may respond differently to irradiation. Cells in the blood and spleen (e.g., mast cells, granulocytes, macrophages, dendritic cells, natural killer cells, B-cells, and stromal cells) may be susceptible to irradiation and ultimately undergo apoptosis, resulting in reduced thymic and splenic mass or cell density.

In conclusion, neural cell injury caused by heavy ion irradiation induces considerable peripheral immune system dysfunction *in vitro* and *in vivo*. This dysfunction can last for a relatively long time. However, irradiation mobilizes the peripheral innate immune system. Our results will facilitate therapeutic manipulation of the immune interface to mitigate the adverse late side effects of radiotherapy in patients with brain cancers. Follow-up studies should identify the types of immune cells responsible for the side effects and potential internal adjustment mechanisms, which will aid the design of bio-therapeutic intervention strategies.

## 4. Experimental Section

### 4.1. Cells and Animals

SH-SY5Y, U87, THP-1 and Jurkat were obtained from Cell Center of Peking Union Medical College (Beijing, China). Fresh or conditioned Minimum Essential Medium (MEM) supplemented with 10% heat-inactivated fetal bovine serum (Gibco, Life Technologies, Carlsbad, CA, USA), 100 units/mL penicillin and 100 μg/mL streptomycin (Beijing Solarbio Science & Technology Co., Beijing, China) was used for cell culture during the irradiation period. For routine culture, DMEM, MEM and Roswell Park Memorial Institute (RPMI)-1640 supplemented with fetal bovine serum and antibiotics were used for SH-SY5Y, U87 and the immune cells, respectively. The culture was maintained at 37 °C in humidified incubator containing 5% CO_2_.

Specific pathogen-free Wistar rats (male; weight, 180 ± 10 g, Vital River Laboratory Animal Technology Co., Ltd., Beijing, China) were maintained under standard conditions in the animal house (12 h light/12 h dark cycle; temperature, 20–26 °C; humidity, 40%–70%) with free access to food and water. All procedures were approved by Beijing Institute of Technology and Key Laboratory of Heavy Ion Radiation Biology and Medicine of Chinese Academy of Sciences.

### 4.2. Carbon Ion Radiation Procedure and Experimental Design

SH-SY5Y, U87 and their combination (1:1) were seeded in T25 cell culture flask at a density of 4.5 × 10^5^ with 5 mL medium. After overnight incubation for cell adhesion, cells were irradiated in the horizontal direction at the dose of 1 Gy, 2 Gy and 5 Gy (165 MeV/u primary energy; liner energy transfer (LET), 30 KeV/μm; intensity, 0.3–0.5 Gy/min) at Heavy Ion Research Facility in Lanzhou (HIRFL), Institute of Modern Physics, Chinese Academy of Sciences, Lanzhou, China. An immediate replacement of equal volumes fresh medium was performed after irradiation. Twenty-four hours after irradiation, conditioned medium was collected after 2000× *g* centrifugation for 10 min. Then, the medium was used for continuous immune cell culture and cell viability assay.

Rats were randomly divided into control and BLCIR groups (*n* = 42, 21 rats per group, seven rats per time point). All rats were weighed, anesthetized via intraperitoneal injection of pentobarbital sodium salt solution (40 mg/kg body weight; concentration, 20 mg/mL), and fixed to the irradiation equipment at HIRFL. Rats in the experimental group (*n* = 21) were irradiated vertically on the back of the head with a ^12^C^6+^ ion beam (165 MeV/u primary energy; LET, 30 KeV/μm; intensity, 0.3–0.5 Gy/min). Exposure of the eyes to carbon ion radiation can result in blindness, which impedes rat survival. Thus, the area of the brain receiving radiation covered the olfactory bulb trailing edge to the spinal cord (1.5 cm length). The total absorbed dose was 15 Gy. The accuracy and uniformity of beam was confirmed by the staff in HIRFL. Rats in the control group were anesthetized and fixed for the same amount of time without irradiation. Rats were sacrificed one, two, and three months after exposure.

### 4.3. Histopathology and Apoptosis

Rats were weighed, deeply anesthetized (pentobarbital sodium salt, 60 mg/kg body weight; concentration, 20 mg/mL) and sacrificed. After perfusion with cold normal saline, the brain, thymus, and spleen were immediately excised on an ice-cold plate, weighed, and washed with phosphate buffer solution in preparation for subsequent experiments. Whole brain, thymus and spleen tissues of three randomly selected rats in each group were fixed with 4% paraformaldehyde, dehydrated, and embedded in paraffin following a standard procedure. Tissue sections (brain, 8 μm thickness; thymus and spleen, 4 μm thickness) were stained with Hematoxylin and eosin staining for histopathological examination. Apoptotic cells in the thymus and spleen were detected via TUNEL method following the instructions in the kit (Millipore, MA, USA, #S7101). A quantitative evaluation of the number of specialized neural cells, Lyons blue and TUNEL-positive cells in the sections was carried out using unbiased stereological counting methods. Five different views on each section with a magnification of ×400 were counted.

### 4.4. Enzyme-Linked Immunosorbent Assay (ELISA)

Amounts of inflammation factors SSAO, TNF-α, IL-6 and HMGB1 in peripheral blood serum, and IL-10 in spleen homogenates were measured using ELISA kits according to the manufacturer's instructions. Kit for HMGB1 was purchased from Global Biotech Co., Ltd. Shanghai, China (#ABIN367992), and the others were acquired from Beijing Hankehengyu Bio-Technology Co., Ltd. Beijing, China.

### 4.5. Real-Time Quantitative PCR

Real-time quantitative PCR assessing changes in the levels of mRNAs encoding proteins involved in T lymphocyte development was performed as previously described [57]. The primers for c-kit, Sca1, Rag1, Rag2 and GAPDH are listed in supplementary materials (Table S1). Data were analyzed using iQ5 Optical System software, version 2.1 (Bio-Rad Laboratories, Inc., Hercules, CA, USA). Samples from four randomly selected rats in each group were assayed.

### 4.6. Flow Cytometry Analysis of T Lymphocyte Subsets

Flow cytometry was performed according to the manufacturer’s instructions. Freshly prepared single cell suspensions of blood, thymus and spleen samples from seven animals per group were incubated with anti-rat CD3, CD4, or CD8 antibodies. Event Count: 100,000. The percentage of CD3^+^ T cell subset in peripheral blood and thymus was normalized to that of total lymphocyte, respectively. In thymic and splenic T lymphocyte subsets analysis, the number of CD3^+^ T-cells was used to normalize. All antibodies were purchased from Bio-Legend (San Diego, CA, USA). Stained cells were analyzed using a FACSCalibur flow cytometer (BD Biosciences, San Jose, CA, USA) and Cell Quest software (BD Biosciences).

### 4.7. Complete Blood Count of Peripheral Blood

Hematological indices in 15 μL peripheral blood of each sample were determined using an automated hemocytometer (pocH-VD, Laboman easyAccess Software 4.0, IDEXX Laboratories (Shanghai) Co., Ltd.) immediately after blood was gently pumped from the left cardiac ventricle of rats. Lymphocytes in blood were presented.

### 4.8. Cell Viability Assay

Cell viability was evaluated by using CellTiter 96^®^ AQ_ueous_ One Solution Cell Proliferation Assay according to the manufacturer’s instructions (Cat. G3581, Promega Corporation, an affiliate of Promega (Beijing) Biotech Co., Ltd.). Briefly, 6000 immune cells with 100 μL conditioned medium were seeded in each well on 96-well plate. 24 h later, 20 μL of provided regents were added in each well and then incubated at 37 °C for 2 h before reading in a fluorometer at 490 nm. The optical density (OD) was given to evaluate cell viability. At least six duplications were set.

### 4.9. Cell Migration Assays

The migration of THP-1 cells was evaluated using a 6.5 mm diameter and 3.0 μm pore polystyrene plates (#3415, Costar, Corning, NY, USA). THP-1 cells were washed twice with serum-free medium and resuspended in serum-free medium. 200 μL containing 2 × 10^5^ cells were added to the upper chamber of the apparatus. The lower chamber contained 600 μL conditioned medium. Twenty-four hours after incubation, the migrated cells were fixed with 4% paraformaldehyde and then stained for 25 min with giemsa. The filters were washed five times with ddH_2_O and the non-migrating cells were then carefully removed from the upper surface (inside) of the transwell with a wet cotton swab. Cells that had migrated or invaded to the bottom surface of the filter were counted. Ten random fields (×400 magnification) were captured for each membrane, and the migrated cells were counted.

### 4.10. Statistical Analysis

The data were expressed as mean ± standard deviation (SD). Analysis of variance with multiple comparison tests was used to determine the significance of differences between groups. *p* < 0.05 indicated a statistically significant difference. Correlation statistical analyses were performed using GraphPad, version 5.01 (GraphPad Software, Inc., La Jolla, CA, USA). Brain Explorer software version 2.3 (Allen Institute for Brain Science, Seattle, WA, USA) was used to model the rat brain.

## Supplementary Materials

Supplementary materials can be found at http://www.mdpi.com/1422-0067/16/12/26109/s1. Primers’ sequence (Table S1); Level of adrenocorticotropic hormone and corticotrophin-releasing hormone (Figure S1); Level of glucocorticoids and glucocorticoid receptors (Figure S2).

## Abbreviations

BLCIR	brain-localized carbon ion radiation
SSAO	semicarbazide-sensitive amino oxidase
TNF-α	tumor necrosis factor-α
IL-6	interleukin 6
HMGB1	high mobility group box-1
NS	not significant
LYM	lymphocyte
TUNEL	terminal deoxynucleotidyltransferase dUTP nick end labeling
PCR	polymerase chain reaction
IL-10	interleukin 10
HPA	hypothalamic-pituitary-adrenal
GR	glucocorticoid receptor
GC	glucocorticoid
FBS	fetal bovine serum
HIRFL	Heavy Ion Research Facility in Lanzhou
LET	liner energy transfer
ELISA	enzyme-linked immunosorbent assay
