# Supplementary material for: Carbon Ion Irradiated Neural Injury Induced the Peripheral Immune Effects in Vitro or in Vivo
Source: Int J Mol Sci. 2015 Nov 30;16(12):28334–46. doi: 10.3390/ijms161226109 (PMC4691056; doi:10.3390/ijms161226109)
Supplement: Supplementary file 1 [file ijms-16-26109-s001.pdf]

Supplementary Materials: Carbon Ion Irradiated Neural Injury Induced the Peripheral Immune Effects *in Vitro* or *in Vivo*

Runhong Lei, Tuo Zhao, Qiang Li, Xiao Wang, Hong Ma and Yulin Deng

Table S1. Primers' sequence.

Name	Forward (5′-3′)	Reverse (5′-3′)
c-kit	GATGCTCAAACCAAGTGCCC	GCGCCAAGCAGGTTCACAAT
Sca1	ACCCCTATGAGTCCAGGCAT	CCGCTCTTGTCATTGAGGGT
Rag1	CCCGTGGACGCTAAAACTCA	ATCTGCCTTCACGTCGATCC
Rag2	TCAGGACGGGCTGTCTTTTC	TGCAATTCACTGCTGGGGTA
GAPDH	CTCATGACCACAGTCCATGC	TTCAGCTCTGGGATGACCTT

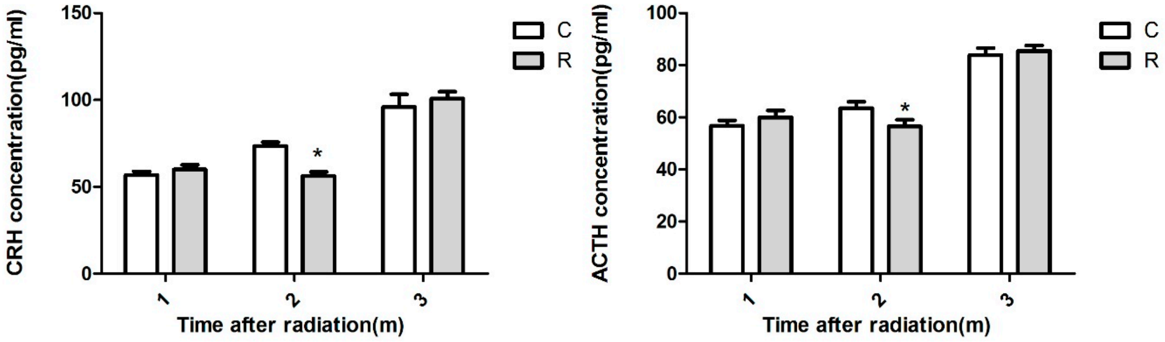

Figure S1. Concentrations of corticotrophin-releasing hormone (CRH) and adrenocorticotropic hormone (ACTH) in the peripheral blood. C: control group (mock irradiated). R: Brain-localized radiation group. m: month. $n = 7$. * $p < 0.05$.

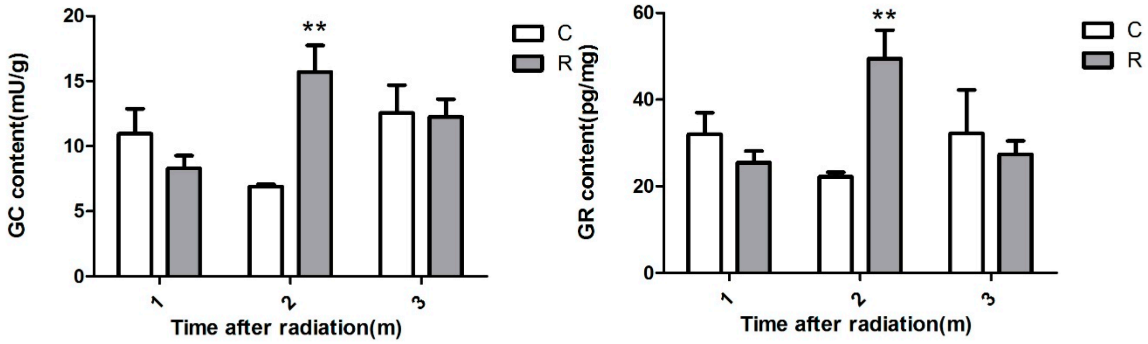

Figure S2. Concentrations of glucocorticoids (GC) and glucocorticoid receptor (GR) in thymus. C: control group (mock irradiated). R: Brain-localized radiation group. m: month. $n = 7$. ** $p < 0.01$.
